# Intrathecal Administration of High-Titer Cytomegalovirus Immunoglobulin for Cytomegalovirus Meningitis

**DOI:** 10.1155/2014/272458

**Published:** 2014-05-08

**Authors:** Shin-ichiro Fujiwara, Kazuo Muroi, Raine Tatara, Ken Ohmine, Tomohiro Matsuyama, Masaki Mori, Tadashi Nagai, Keiya Ozawa

**Affiliations:** Division of Cell Therapy and Hematology, Jichi Medical University Hospital, 3311-1 Yakushiji, Shimotsuke, Tochigi 329-0498, Japan

## Abstract

Cytomegalovirus (CMV) central nervous system disease after hematopoietic stem cell transplantation (HSCT) is a rare but life-threatening complication. Here, we report a patient who developed CMV meningitis after HSCT and was treated with the combination therapy of intrathecal high-titer CMV immunoglobulin and antiviral drugs. A 38-year-old man with myelodysplastic syndrome received a cord blood transplant after graft failure. On day 147, he was diagnosed with CMV meningitis based on pleocytosis and CMV DNA in the cerebrospinal fluid (CSF). Intravenous ganciclovir, foscarnet, and immunoglobulin were administered; however, CMV DNA in the CSF was continuously detected. The addition of intrathecal high-titer CMV immunoglobulin resulted in CMV DNA in the CSF becoming undetectable. On day 241, CMV DNA in the CSF was detected again, but both intrathecal immunoglobulin and intravenous ganciclovir led to its disappearance. No adverse effects related to intrathecal administration were observed. The intrathecal administration of immunoglobulin may be safe and effective for CMV meningitis.

## 1. Introduction

CMV disease is still one of the most serious complications after HSCT. By preemptive therapy against CMV reactivation, CMV disease has been successfully reduced during the first several months after HSCT. In contrast, late-onset CMV disease after the completion of monitoring the viral load is now frequently observed in high-risk patients [1]. CMV central nervous system (CNS) disease developing at such a stage has rarely been reported [2, 3]. The occurrence of CMV CNS disease is associated with high mortality due to impaired CMV-specific immunity, antiviral drug resistance, or both [2]. Therefore, new therapeutic strategies for CMV CNS disease are needed.

## 2. Case Presentation

**Figure 1:**
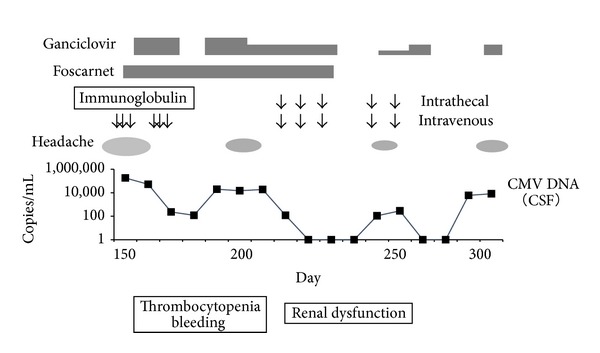
Clinical course of patient. Day means day from second cord blood transplantation. CMV: cytomegalovirus, CSF: cerebrospinal fluid.

A 38-year-old man with myelodysplastic syndrome (refractory anemia with excess blasts-2) received a cord blood transplant. Conditioning was a myeloablative conditioning regimen consisting of cyclophosphamide and total body irradiation. The treatment for the prevention of the graft-versus-host disease (GVHD) was tacrolimus and short-term methotrexate. Cord blood (0.67 × 10^5^ CD34^+^ cells/kg) was infused and granulocyte colony-stimulating factor (G-CSF) was given from day 1. On day 29, the patient suffered from primary graft failure caused by hemophagocytic syndrome. Following nonmyeloablative conditioning including fludarabine (30 mg/m^2^/day, 2 days), etoposide (30 mg/m^2^, 1 day), and antithymocyte globulin (2.5 mg/kg/day, 2 days), cord blood (0.76 × 10^5^ CD34^+^ cells/kg) was infused again [4]. GVHD prophylaxis was tacrolimus alone (0.03 mg/kg/day, continuous infusion from day 1), and G-CSF was given from day 1. On day 16, from the second cord blood transplantation, meningitis due to* Enterococcus faecalis* developed. The administration of intravenous linezolid and intrathecal vancomycin resulted in the negativity of the bacteria in the cerebrospinal fluid (CSF). Neutrophilic engraftment was achieved on day 46. After engraftment, CMV antigenemia was repeatedly positive, and preemptive ganciclovir and foscarnet were effective for CMV viremia. CMV retinitis developed on day 96 and ganciclovir led to an improvement of fundus lesions due to CMV. There were no signs of GVHD, and tacrolimus was discontinued on day 137. From day 138, he complained of headache, high fever, and ocular pain. Magnetic resonance imaging of the brain did not show encephalitis. On day 147, a lumbar puncture revealed meningitis based on an increased number of CSF cells (404 cells/mm^3^), increased level of CSF protein (148 mg/dL), and decreased level of CSF glucose (39 mg/dL). The CMV DNA level in the CSF was 1.8 × 10^5^ copies/mL measured with a qualitative polymerase-chain-reaction method, and the patient was diagnosed with CMV meningitis. Ganciclovir, foscarnet, and immunoglobulin were intravenously administered, but it should be noted that ganciclovir must be stopped in the presence of a bleeding tendency and thrombocytopenia. CMV DNA in the CSF was continuously detected and headache developed again on day 201 (Figure 1). The dose of ganciclovir must have been reduced by myelosuppression. Neurological symptoms caused by CMV meningitis worsened progressively. Therefore, after obtaining permission from the division director and consent from the patient, high-titer CMV immunoglobulin (hCMV-IG: complete-type immunoglobulin, Kenketsu Venilon-I, Kaketsuken, Kumamoto, Japan) was intrathecally administered from day 208. Firstly, 0.25 g taken from a 5 g of hCMV-IG was administered intrathecally and the remaining 4.75 g was administered intravenously. Subsequently, 0.5 g of hCMV-IG was administered intrathecally and 4.5 g of the agent was administered intravenously [5, 6]. Intrathecal hCMV-IG administration was performed weekly until day 223 (a total of 3 doses). No adverse effects related to the intrathecal administration of hCMV-IG, such as neurological symptoms and allergic reactions, were observed. The intrathecal IgG level (mean 27, range 25–28 mg/dL) was lower than serum IgG (mean 923, range 900–960 mg/dL). Following the intrathecal administration of hCMV-IG, CMV DNA became undetectable and ganciclovir and foscarnet were discontinued. Although headache and CMV DNA in the CSF reappeared on day 241, the administration of intrathecal hCMV-IG (a total of 2 doses) and intravenous ganciclovir resulted in its disappearance. No adverse effects associated with hCMV-IG administration were observed. Although the number of neutrophils and immunoglobulin levels recovered to normal range, cellular immune deficiency continued after the second transplantation. The number of lymphocytes, CD4 cells, and CD8 cells remained at less than 500, 100, and 100/mm^3^, respectively. CMV-specific cytotoxic T lymphocytes were not detected on a tetramer assay after hCMV-IG intrathecal administration (data not shown). CMV meningitis worsened from around day 300, and it was difficult to continue treatment due to deterioration of the patient's general condition. The patient died of sepsis on day 338, and an autopsy was not performed.

## 3. Discussion

Immunoglobulin available in Japan can be categorized into two types: incomplete and complete. The former is a disconnected Fc region, while the latter has an intact Fc region. Intrathecal administration of the incomplete type has been approved in Japan. Regarding the complete type, there have been several reports on intrathecal administration for refractory bacterial meningitis and other conditions [5–8]. To our knowledge, ours is the first reported case where CMV meningitis was treated with the combination therapy of intrathecal hCMV-IG and antiviral drugs. In our case, one or two doses of intrathecal hCMV-IG administration led to the clearance of CMV DNA in the CSF. The initial response may have simply reflected the effects of ganciclovir and foscarnet. However, the long-term administration of either ganciclovir or foscarnet or both is impossible due to their toxic effects such as causing myelosuppression and renal dysfunction. Intrathecal immunoglobulin administration may be worth trying, along with an anti-CMV agent, for an immediate response in CMV meningitis patients.

hCMV-IG administered intrathecally may neutralize cell-free CMV, inhibit cell-to-cell virus spread, and reduce CMV mRNA levels in infected cells [8]. In addition to such direct effects, intrathecally administered immunoglobulin may neutralize inflammatory cytokines aggravating meningitis, such as interleukin-1 and tumor necrosis factor, as shown by dexamethasone for bacterial meningitis [9]. In our case, as reported previously [5, 6], 0.5 g of complete-type immunoglobulin was safe and effective when administered intrathecally. Further studies are needed to determine the optimal dose of intrathecal immunoglobulin.

The effect of intrathecal hCMV-IG did not persist for a long time. Surviving patients with CMV CNS disease showed cellular immune recovery against CMV [2, 3]. Therefore, CMV-specific T cells are essential in the control of CMV disease. In our case, CMV-specific T cells were absent throughout the course after second cord blood transplantation. The absence of cellular immunity may have resulted in a short-term effect of hCMV-IG against CMV meningitis. Recently, the clinical use of CMV-specific cytotoxic T cells against CMV disease has progressed [10]. The combination of intrathecal hCMV-IG administration and such adoptive immune cell therapy may be meaningful for CMV CNS disease in immunocompromised patients after HSCT.

In conclusion, we are the first to demonstrate that the combination of intrathecal hCMV-IG and antiviral drugs is safe and effective for CMV meningitis. Because CMV CNS disease is still fatal, intrathecal hCMV-IG administration may be a treatment option in addition to antiviral therapy and adoptive immune cell therapy.

## References

[1] Asano-Mori Y, Kanda Y, Oshima K (2008). Clinical features of late cytomegalovirus infection after hematopoietic stem cell transplantation. *International Journal of Hematology*.

[2] Reddy SM, Winston DJ, Territo MC, Schiller GJ (2010). CMV central nervous system disease in stem-cell transplant recipients: An increasing complication of drug-resistant CMV infection and protracted immunodeficiency. *Bone Marrow Transplantation*.

[3] Tam DY, Cheng FW, Chan PK (2012). Intact survival of refractory CMV limbic encephalitis in a patient with severe aplastic anemia after unrelated bone marrow transplantation. *Journal of Pediatric Hematology/Oncology*.

[4] Sumi M, Shimizu I, Sato K (2010). Graft failure in cord blood transplantation successfully treated with short-term reduced-intensity conditioning regimen and second allogeneic transplantation. *International Journal of Hematology*.

[5] Nishizawa E, Yamamoto H, Kawano T, Kamata K (1988). The clinical study of intrathecal administration of immune globulin against meningitis. *The Journal of Therapy*.

[6] Tokito S, Ohyama M, Motoghishita T, Yamashita H (1981). Experience of intrathecal administration of Venilon in neurosurgery area. *Medical Consultation & New Remedies*.

[7] Watanabe H, Hasegawa T, Saito T (1984). Intrathecal and intravenous combined administration of Venoglobulin—I for meningitis. *Journal of New Remedies & Clinics*.

[8] Kudoh C, Ikegami Y, Sugiura K (1995). Combined treatment of pH4 treated acidic human normal immunoglobulin and antibiotics for severe infections in a neurosurgical patients. *Japanese Journal of Neurosurgery*.

[9] Lutsar I, Friedland IR, Jafri HS (2003). Factors influencing the anti-inflammatory effect of dexamethasone therapy in experimental pneumococcal meningitis. *Journal of Antimicrobial Chemotherapy*.

[10] Sellar RS, Peggs KS (2012). Therapeutic strategies for the prevention and treatment of cytomegalovirus infection. *Expert Opinion on Biological Therapy, Informa Healthcare*.

